# Is the Cally index truly a prognostic marker that predicts mortality in older patients with acute coronary syndrome?

**DOI:** 10.3389/fcvm.2026.1837291

**Published:** 2026-07-23

**Authors:** Ebru Serin, Mert Sarılar, Sinan Şahin, Arzu Er Kara, Hasan Değirmenci, Erol Kalender, Ahmet Gürdal, Kudret Keskin

**Affiliations:** Department of Cardiology, Şişli Hamidiye Etfal Training and Research Hospital, Istanbul, Türkiye

**Keywords:** acute coronary syndrome, biomarker, CALLY index, mortality, older patients

## Abstract

**Background:**

The C-reactive protein (CRP) -albumin- lymphocyte (CALLY) index has recently emerged as a promising prognostic marker, which reflecting inflammatory, nutrional and immunological statuses. However, no study has reported the prognostic value of the CALLY index in older patients with acute coronary syndrome (ACS). The aim of the study is to determine whether the CALLY index can serve as a reliable predictor of all- cause mortality in older patients with ACS.

**Methods:**

This study was conducted retrospectively in patients aged ≥ 75 years old with ACS. The study comprised 440 patients diagnosed with ACS at our hospital May 2015 to August 2024. The median follow-up time was 23 months. The CALLY index was computed using the formula: (serum albumin (g/dL)×lymphocyte (10^9^/L)/CRP (mg/L)×10). The primary outcome was all-cause mortality from the day of admission.

**Results:**

A total of 475 ACS patients over 75 years old were ultimately enrolled in this study. 35 patients were excluded due to missing follow-up data and active infection. The patients were divided into two groups according to the CALLY index: low CALLY (≤ 0.083, n:219), high CALLY (> 0.083, n:221). The low CALLY index appears to be a valuable tool for predicting mortality in older patients with ACS. Kaplan–Meier analysis demonstrated that a low CALLY index was significantly associated with low survival rate. Our study revealed that the CALLY index provides statistically significant independent predictor in older patients' prognosis in all-cause mortality. All- cause mortality was 48.2% (n:212) in both groups. In the low CALLY index group, a higher rate of all- cause mortality was observed [group 1, n:130 (59.4%)].

**Conclusions:**

Based on the findings of our study, the CALLY index appears to be a practical and easily applicable tool for assessing mortality risk in elderly patients with acute coronary syndrome.

## Introduction

Coronary artery disease (CAD) remains a major global health problem with an increasing prevalence, posing significant challenges not only to patients' physical and psychological well-being but also to healtcare systems due to its substantial economic burden. Among individuals presenting with ACS, particularly in elderly population, clinical outcomes are strongly influenced by underlying inflammatory, nutritional, and immunological conditions, which may contribute to the development of complications during recovery.

Many studies have examined the combined use of inflammatory markers [systemic immün inflammatory index (SII), Naples prognostic score, etc.] in patients who have CAD (1). However, the sensitivity value of these markers in daily diagnosis can vary. Although the availability of many predictive inflammatory biomarkers for mortality in CAD has been shown in studies, few are effective, particularly in older patients with ACS.

Inflammation plays a central role in the pathophysiology of ACS. The progression of atherosclerosis involves complex interactions between inflammatory process and plaque instability, ultimately leading to thrombus formation. In the atherosclerotic process, a variety of triggers act on vulnerable plaques, and thrombus formation with features of inflammation.

The CALLY index is a biomarker that combines serum albumin, lymphocytes, and CRP to determine a patients' prognosis. Recent studies have identified a significant negative linear relationship between the CALLY index and cardiovascular disease (CVD) mortality in elderly patients (2). Although few studies have been reported to assess the relationship between the CALLY index and cardiovascular events, there is no further research focusing on the relationship between the CALLY index and ACS in older patients (3–6).

In this retrospective study aimed to explore the prognostic significance of the CALLY index in elderly patients diagnosed with ACS and to clarify its potential role as a predictor of clinical outcomes in this high-risk population.

## Materials and methods

### Study population

The study was conducted in accordance with the Declaration of Helsinki (as revised in 2013). This study received approval from the Ethics Committee of Seyrantepe Hamidiye Etfal Training and Research Hospital. Patients did not provide written informed consent due to the retrospective study design.

**Figure 1:**
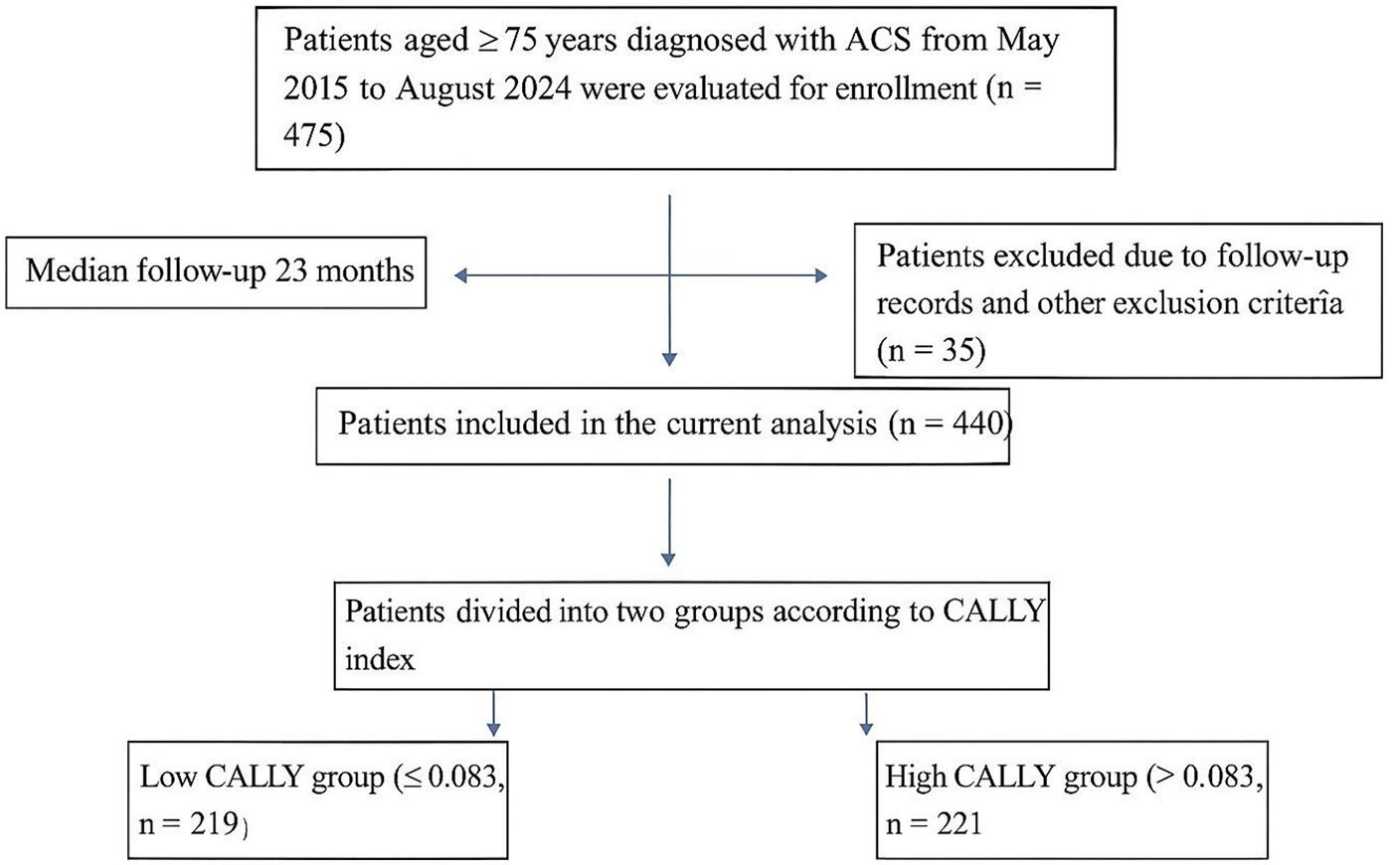
Flowchart of the study.

From May 2015 to August 2024, at the Seyrantepe Hamidiye Etfal Training and Research Hospital Cardiology Department, was examined for 475 ACS patients over 75 years old. A total of 35 patients were excluded due to follow-up records, and other exclusion criteria (Figure 1). To ensure data integrity and eliminate potential confounding effects on systemic inflammatory, nutritional, and immunological biomarkers, a rigorous selection process was applied to the study population. Regarding the management of missing data, a complete-case analysis approach was employed; patients lacking baseline laboratory measurements required to compute the CALLY index, or those with incomplete electronic medical and telephone follow-up records, were comprehensively excluded from the final cohort without the use of statistical imputation techniques. Furthermore, patients presenting with the following clinical conditions were meticulously excluded: active severe infections cardiogenic shock, cardiac surgery requiring urgent coronary revascularization, severe valvular heart disease, prosthetic heart valves, pulmonary embolism, presence autoimmune or systemic inflammatory diseases, life-threatening bleeding, coagulation abnormalities, advanced hepatic failure (Child– Pugh class C), severe renal failure, trauma, recent major surgery, active malignancy, hypo/hyperthyroidism, or death during follow-up.

Regarding the management of missing data, a complete-case analysis approach was employed. Patients with missing baseline laboratory measurements required to compute the CALLY index, or those lacking complete electronic medical and telephone follow-up records, were comprehensively excluded from the final cohort. No statistical imputation techniques were utilized for missing variables.

After applying exclusion criteria, we examined 440 eligible patients.

ACS was described based on the 2023 Europian Society Cardiology (ESC) guidelines for the management of ACS (7). Information on laboratory examinations, demographic data, morbidity, and mortality were meticulously gathered through telephone interviews and electronic medical records database of our hospital.

In this study, the formula of the CALLY is: Serum albumin (g/dL)×Lymphocyte (10^9^/L)/(CRP (mg/L) ×10). The biochemical and hematological laboratory results from the venous blood taken at the first visit to the hospital.

440 patients who had enrolled in this analysis were divided into two groups according to the CALLY index: low CALLY (≤ 0,083, n:219), high CALLY (> 0.083, n:221) index groups. Receiver operating characteristic (ROC) curve analysis was used to assess the predictive performance of the CALLY index for all-cause mortality. The optimal cut-off value was determined using the Youden index. The occurrence of all cause- mortality was the primary endpoint.

### Statistical analysis

All statistical analyses were performed in SPSS, version 25.0 (IBM Inc., Armonk, NY, USA), and a *p* value of less than 0.05 was considered statistically significant. Kolmogorov –Smirnov tests were performed to assess the normality of data distribution. Categorical variables were described as counts (percentage), and continuous variables were expressed as medians (25th– 75th percentiles). A non-normal distribution of variables was assumed based on the sample size, and non-parametric tests were used for between-group comparisons. Comparisons between groups for categorical data were made using the continuity-corrected chi-squared statistic of Pearson's chi-squared test. Continuous variables were compared between groups using the Mann–Whitney U tests. Spearman's test evaluated the correlation between the CALLY index and some different variables. To determine the independent predictors of mortality and control for potential confounding clinical factors, univariate Cox regression analyses were initially performed including age, gender, diabetes mellitus (DM), creatinine level, LVEF %, CALLY index, systemic immune-inflammation index (SII), LMR, PLR and hemoglobin level as covariates. Variables demonstrating a *p*-value < 0.1 in the univariate analysis were subsequently entered into a multivariate Cox proportional hazards regression model to adjust for these baseline confounders. Variables were examined at the 95% confidence level. The prognostic difference and event-free survival rate between patients with CALLY index groups were analyzed using the Kaplan–Meier method, and the significance was evaluated using log-rank tests. Probability (p) values of <0.05 were considered statistically significant.

## Results

A total of 440 elderly patients with ACS, divided into two groups according to the CALLY index (≤ 0.083, n:219;>0.083, n:221). The median age was 81 (77–85) years. 229 (52%) patients were man. The observed all -cause mortality rates were 59.4% in the low- CALLY group and 37.1% in the high- CALLY group. The baseline characteristics of the patients according to the CALLY index are demonstrated in Table 1.

**Table 1:** Baseline clinical, comorbidity, and laboratory characteristics of study population according to CALLY Index categories.

Variables	Overall (*n* = 440)	CALLY ≤0.083 (*n* = 219)	CALLY >0.083 (*n* = 221)	*P*-value
Demographics				
Age, years	81 (77–85)	82 (77–85)	80 (77–84)	0.313
Male sex, *n* (%)	229 (52.0)	120 (54.8)	109 (49.3)	0.254
Comorbidities				
Hypertension, *n* (%)	327 (74.3)	165 (75.3)	162 (73.3)	0.663
Dyslipidemia, *n* (%)	143 (32.5)	62 (28.3)	81 (36.7)	0.067
Diabetes mellitus, *n* (%)	174 (39.5)	95 (43.4)	79 (35.7)	0.119
History of CAD, *n* (%)	149 (33.9)	70 (32.0)	79 (35.7)	0.421
History of CVD, *n* (%)	39 (8.6)	18 (8.2)	20 (9.0)	0.866
Atrial fibrillation, *n* (%)	109 (24.8)	55 (25.1)	54 (24.4)	0.912
Cardiac characteristics				
LVEF (%)	49 (40–60)	45 (35–60)	50 (40–60)	0.013
STEMI, *n* (%)	183 (41.6)	90 (41.1)	93 (42.1)	0.847
UA/NSTEMI, *n* (%)	257 (58.4)	129 (58.9)	128 (57.9)	0.910
Laboratory findings				
Glucose, mg/dL	138 (110–183)	139 (111–184)	136 (110–182)	0.537
Total cholesterol, mg/dL	175 (139–205)	166 (136–198)	182 (148–210)	0.002
HDL cholesterol, mg/dL	41 (35–49)	40 (33–48)	42 (36–50)	0.002
LDL cholesterol, mg/dL	113 (83–138)	104 (78–134)	117 (88–144)	0.001
Triglycerides, mg/dL	115 (86–161)	116 (88–162)	115 (85–160)	0.792
Creatinine, mg/dL	1.1 (0.9–1.4)	1.2 (0.9–1.6)	1.0 (0.8–1.2)	0.001
Hemoglobin, g/dL	12.3 (11.2–13.8)	12.0 (10.6–13.4)	12.7 (11.6–14.0)	0.001
WBC count (× 10³/µL)	9.0 (7.2–11.4)	9.7 (6.6–12.7)	8.4 (7.1–10.7)	0.001
Neutrophil (× 10³/µL)	7.2 (5.3–8.8)	7.2 (5.3–10.2)	5.5 (4.3–7.7)	0.001
Lymphocyte (× 10³/µL)	1.8 (1.2–2.4)	1.5 (1.0–2.0)	2.1 (1.6–2.6)	0.001
Monocyte (× 10³/µL)	0.6 (0.4–0.8)	0.6 (0.5–0.8)	0.5 (0.4–0.7)	0.046
Platelet (× 10⁹/L)	220 (185–269)	229 (192–287)	211 (180–259)	0.097
CRP, mg/L	8.0 (3.0–28.3)	28.7 (14.7–66.9)	3.2 (1.7–5.4)	0.001
Inflammatory and nutritional indices				
SII	866 (460–1,388)	1,126 (714–2,079)	543 (369–834)	0.001
NLR	3.52 (2.20–6.07)	4.28 (2.33–8.38)	2.57 (1.83–3.78)	0.001
LMR	3.13 (2.0–4.5)	2.42 (1.8–3.6)	3.66 (2.8–5.2)	0.001
PLR	126 (91–183)	140 (104–228)	100 (77–138)	0.001
Albumin, g/dL	3.7 (3.2–4.1)	3.5 (3.0–4.0)	3.8 (3.4–4.2)	0.001
Outcome				
Long-term mortality, *n* (%)	212 (48.2)	130 (59.4)	82 (37.1)	0.001

Continuous variables are presented as median (interquartile range), and categorical variables as number (percentage). CALLY, CRP/albumin×lymphocyte ratio; CAD, coronary artery disease; CVD, cerebrovascular disease; STEMI, ST-elevation myocardial infarction; UA/NSTEMI, unstable angina/non-ST-elevation Myocardial Infarction; LVEF, left ventricular ejection fraction; CRP, C-reactive protein; HDL, high-density lipoprotein; LDL, low-density lipoprotein; SII, systemic immune-inflammation Index; NLR, neutrophil-to-lymphocyte ratio; LMR, lymphocyte-to-monocyte ratio; PLR, platelet-to-lymphocyte ratio.

A post -hoc power analysis was performed using a two-tailed test with *α* = 0.05. Given the observed absolute difference of 22.3 percentage points (59.4% vs. 37.1%), the statistical power to detect his effect was 99.7% (n1: 219, n2: 221). The current study exceeds the sample size required for adequate statistical power.

At the end of the follow-up period, 219 (48.2%) patients were in the low CALLY index group, and 221 (51.8%) patients were in the high CALLY index group. There were no significant differences between the two groups in terms of age, gender male, hypertansion (HT), dyslipidemia, history for CAD, diabetes melllitus (DM), atrial fibriation, history for CVD, ST elevation myocardial infarction, unstabil angina, non-ST elevation myocardial infarction, glucose, triglycerides (*p* > 0.05) among the two groups. According to the multivariate Cox regression analysis, several variables emerged as independent predictors of mortality. A higher CALLY index was identified as an independent protective factor against mortality, demonstrating an HR of 0.384 (95% CI:0.201–0.734, p:0.004). Age (HR of 1.074- 95% CI:1.045–1.105, p:0.001), indicating that increased age is associated with a higher risk of mortality. Similarly, Creatinine levels were independently associated with an increased risk of mortality, presenting an HR of 1.185 (95% CI:1.067–1.317, p:0.002). Furthermore, higher LVEF was also independently associated with a lower mortality risk, with an HR of 0.558 (95% CI: 0.945–0.970, p:0.001). Several variables were significantly different, including Total Cholesterol (mg/dL), low-density lipoprotein (LDL-C), highdensity lipoprotein (HDL-C), hemoglobin (g/dL), WBC count, lymphocyte, neutrophil, monocyte, platet, CRP, systemic immune-inflammation index (SII), NLR, lymphocyte to monocyte ratio (LMR), platelet to lymphocyte ratio (PLR)), CALLY index, albumin, all- cause mortality (*p* < 0.05).

The incidence of all-cause mortality in the low CALLY index group was 130 (59.4%), while high CALLY index group was 82 (37.1%). The incidence of all-cause mortality was lower in the high CALLY index group than in low CALLY index group (*p* < 0.001).

ROC curve analysis was performed to evaluate the ability of the CALLY index to predict all-cause mortality. The area under the curve (AUC) was 0.647 [95% confidence interval (CI):0.596–0.698, *p* < 0.001], indicating acceptable discriminative performance.The optimal cut-off value of the CALLY index was identified as 0.083 using the Youden index, which provided the best balance between sensitivity and specifity.

We included CALLY index and other 8 clinical parameters [age, DM, left ventricular ejection fraction (LVEF %), SII, creatinine (mg/dL), hemoglobin (g/dL), LMR and PLR] in a univarite logistic regression analysis, and those with *p* < 0.1 were further included in the multivarite regression analysis (Table 2). The results of univarite logistic regression analysis showed that age, CALLY index, LVEF, SII, creatinine (mg/dL), and PLR were associated with mortality and the results of further multivarite analysis showed that the age, CALLY index, LVEF, creatinine could be used as independent predictors for the diagnosis of mortality.

**Table 2:** Univariate and multivariate Cox regression analysis of variables related to mortality.

Variables	Univariate HR (95% CI)	*P* value	Multivariate HR (95% CI)	*P* value
Age	1.077 (1.047–1.108)	**0** **.** **001**	1.074 (1.045–1.105)	**0**.**001**
CALLY index	0.262 (0.134–0.513)	**0**.**001**	0.384 (0.201–0.734)	**0**.**004**
Diabetes mellitus	0.834 (0.635–1.096)	0.193		
LVEF (%)	0.954 (0.942–0.966)	**0**.**001**	0.958 (0.945–0.970)	**0**.**001**
SSI	1.002 (1.001–1.004)	**0**.**001**	1.001 (1.000–1.003)	0,400
Creatinine (mg/dL)	1.209 (1.102–1.326)	**0**.**001**	1.185 (1.067–1.317)	**0**.**002**
Hemoglobin (g/dL)	0,982 (0.918–1.051)	0.599		
LMR	0.984 (0.937–1.034)	0.528		
PLR	1.001 (1.00.-1.002)	**0**.**001**	1.000 (0.999–1.002)	0.615

Bold numbers represent the statistical significance.

LVEF, left ventricular ejection fraction; SIl, systemic immune-inflammation index: PLR, platelet to lymphocyte ratio; LMR, lymphocyte to monocyte ratio: HR, hazard ratio; Cl, confidence interval.

The results of Spearman correlation analysis are shown in Table 3. The CALLY index demonstrated a negative correlation with SII, PLR, Neutrophil to lymphocyte ratio (*N*LR) and creatinine (mg/dL). Furthermore, we observed that the CALLY index had a positive correlation with LMR, hemoglobin, total cholesterol (mg/dL).

**Table 3:** Correlation between CALLY index and some different variables.

Variable	Spearman's Rho	*p* value
SIl	−0.538	0.001
PLR	−0.532	0.001
NLR	−0.568	0.001
Creatinine (mg/dL)	−0.268	0.001
LMR	0.450	0.001
Hemoglobin (g/dL)	0.238	0.001
Total Cholesterol(mg/dL)	0.226	0.001

SIl, systemic immune-inflammation index; NLR, neutrophil to lymphocyte ratio; PLR, platelet to lymphocyte ratio; LMR, lymphocyte to monocyte ratio.

**Figure 2:**
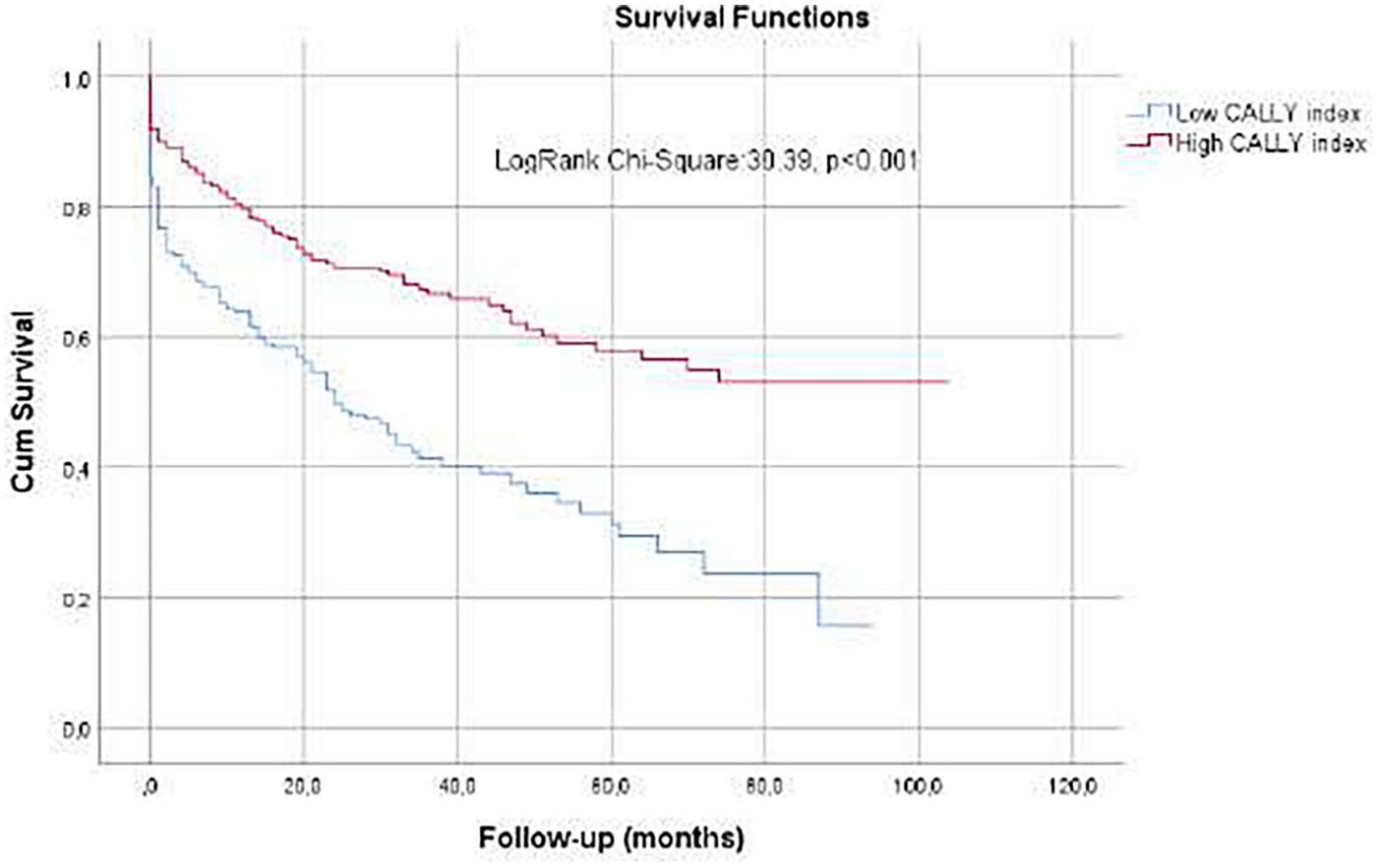
Survival analysis of patients with low CALLY index compared to patients with high CALLY index.

**Figure 3:**
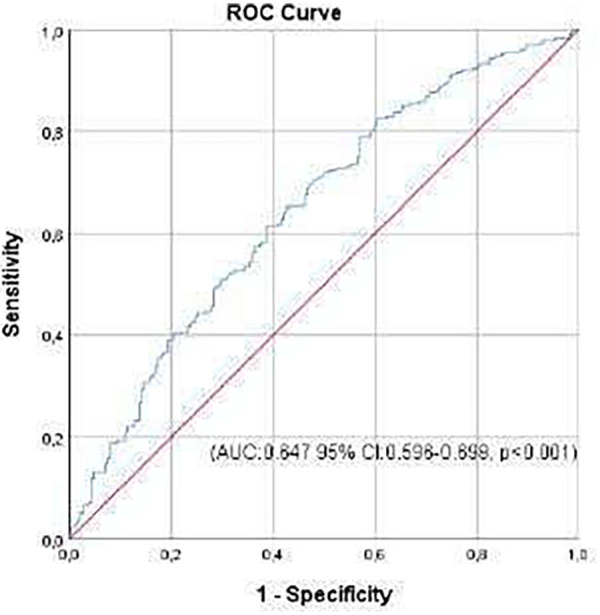
Receiver operating characteristic (ROC) curve for CALLY index. The optimal cut–off value of CALLY index for predicting of mortality was 0.083 with a sensitivity of 61.4% and a specificity of 62,3% (AUC: 0.647 95% CI:0.596–0.698, *p* < 0.001).

Kaplan–Meier curves for the CALLY index and outcomes are shown in Figure 2. To assess the predictive power of CALLY for the prediction of long-term mortality, we computed AUC values, as illustrated in Figure 3. The receiver operating characteristics (ROC) curve demonstrated that the CALLY index holds certain predictive value for mortality risk and clinical outcomes of this population. Collectively, these findings indicate that the CALLY index demonstrates superior discriminatory power and precision in predicting ACS in older patients.

## Discussion

The present study demonstrates a clear association between the CALLY index and all-cause mortality in patients aged 75 years and older presenting with ACS. In this study, lower CALLY index levels are associated with higher mortality risk, while higher CALLY index levels show only a minimal increase in all-cause mortality risk. This relationship may be explained by the individuals component of the CALLY index, as decreased albumin and lymphocyte levels together with elevated CRP reflect a state of heightened inflammation and poor nutritional and immunological status, all of which may contribute to adverse outcomes. As acute-phase reactants, CRP and albumin play opposing roles in the inflammatory response; CRP levels tent to increase during inflammation, whereas albumin levels decline (8). This imbalance reflects systemic inflammatory activation and has been associated with disease severity. We conducted a univariate - multivariate Cox regression analysis, in which, we found that the lower CALLY index was associated with poor prognosis in ACS patients over 75 years old.

In 1994, Liuzzo et al. (9) first determined that high CRP levels may predict poor prognosis for those who had ACS. Elevated serum CRP, produced by the liver in response to inflammatory stimuli, serves as a marker of systemic inflammation (10) and may also directly participate in the inflammatory process of atherogenesis (11). It can delay endothelial repair mechanisms, reduce fibrinolytic activity, reinforce collagen degradation in immune cells, and potentially strengthen LDL uptake by macrophages, turning them into foam cells (12).

Hypoalbuminemia is commonly associated with conditions such as inflammation, malnutrition, and cachexia (13). Reduce serum albumin levels may contribute to progression and severity of CAD through several pathophysiological mechanisms, including increased blood viscosity, endothelial dysfunction, enhanced platelet activation and aggregation, and upregulated production of platelet-derived growth factor (PDGF), which plays a critical role in the development of coronary artery stenosis (14, 15). Furthermore, existing evidence indicates that hypoalbuminemia is an independent predictor of poor clinical outcomes in patients with heart failure and CAD (16–18).

Lymphopenia represents another important component of the CALLY index and is often associated with elevated levels of proinflammatory cytokines, as well as increased cortisol and catecholamine activity (19, 20). In addition, apoptosis of lymphocytes has been observed within atherosclerotic plaques, with its prevalence rising as atherosclerotic disease progresses (21). The redistribution of T cells from the peripheral circulation to lymphoid tissues may further aggravate lymphopenia. This process can stimulate the compensatory expansion of antigen-experienced T-cells, thereby contributing to an elevated risk of cardiovascular events (22). Collectively, these findings were provided theoretical and clinical support for our results.

The cut-off value of 0.083 was derived from ROC analysis within our cohort; however, given the absence of a universally established threshold for the CALLY index, this value should be interpreted with caution.

Therefore, the CALLY index, based on serum albumin, CRP and lymphocyte levels, is a statistically significant independent prognostic biomarker for patients who aged ≥ 75 years old with ACS. The CALLY index is aesily applicable, cost-effective, rapid predictor for clinical practice and independently associated with all- cause mortality in ACS patients.

However, several limitations should be acknowledged. Firstly, the retrospective nature of this study inherently introduces potential selection bias and limits our ability to establish definitive causal relationships. Because of our cohort was drawn from a single institution, the results may not fully represent the wider population of older patients with ACS. We were unable to evaluate MACE as individual endpoints. Furthermore, baseline frailty index scores could not be formally assessed, as comprehensive data regarding patients' functional status and psychosocial variables were not systematically documented in the electronic medical records. Finally, inflammatory and nutritional biomerkers were measured only once at admission, and their temporal variations were not evaluated. Although the 0.083 cutoff represents a reasonable, data- driven threshold with practical clinical relevance, the prognostic performance of CALLY remains limited.

Dichotomizing a continuous variable inevitably results in loss of information; the CALLY index is best utilized as a continuous prognostic measure, with the threshold serving only as a pragmatic decision aid. The 0.083 CALLY threshold provided a statistically discrimination for all- cause mortality, its predictive performance should be interpreted with caution.

A notable limitation of the present study is that the incrementel prognostic value of the CALLY index over established risk scores and conventional biomarkers was not formally evaluated. While we did not incorporate scores like GRACE or SYNTAX in this preliminary retrospective analysis, future prospective, multicenter studies are required to determine the incrementel prognostic value of the CALLY index when adjusted for these established clinical parameters. It is essential to determine whether the CALLY index provides additional discriminatory or predictive value beyond established standarts.

The CALLY index, which integrates parameters reflecting systemic inflammation, nutritional status, and immune competence, may theoretically offer a more comprehensive assessment of patient risk compared to single biomarkers. However, without direct comparative analysis- such as multivariable models including established risk scores, net reclassification improvement (NRI), or integrated discrimination improvement (IDI)- it remains unclear whether the CALLY index contributes meaningful incremental prognostic information.

Future studies incorporating the CALLY index into multivariable models alongside established risk predictors, and evaluating improvements in model performance, would help clarify its potential clinical utility.

In conclusion, although the findings of this study suggest that the CALLY index may be associated with clinical outcomes, its incremental value over established risk models has not been demonstrated and warrants further investigation in larger, prospective cohorts.

Consequently, these results should be interpreted as hypothesis-generating. Given that this study was conducted within a single-center cohort, further validation of these findings is required through larger, multicenter investigatitions involving more diverse patient populations.

## Conclusion

The CALLY index holds certain moderate predictive prognostic value for all-cause mortality risk and clinical outcomes in ACS patients. Incorporating the CALLY index into clinical practice may assist clinicians in identifying high-risk elderly patients and support earlier, more tailored therapeutic decision-making.

## Data Availability

The raw data supporting the conclusions of this article will be made available by the authors, without undue reservation.
